# Entropy Related to *K*-Banhatti Indices via Valency Based on the Presence of *C*_6_*H*_6_ in Various Molecules

**DOI:** 10.3390/molecules28010452

**Published:** 2023-01-03

**Authors:** Muhammad Usman Ghani, Francis Joseph H. Campena, Muhammad Kashif Maqbool, Jia-Bao Liu, Sanaullah Dehraj, Murat Cancan, Fahad M. Alharbi

**Affiliations:** 1 Institute of Mathematics, Khawaja Fareed University of Engineering & Information Technology, Abu Dhabi Road, Rahim Yar Khan 64200, Pakistan; 2 Department of Mathematics and Statistics, College of Science, De La Salle University, 2401 Taft Avenue, Manila 1004, Philippines; 3 The Government Sadiq Egerton College Bahwalpur, University Chowk, Bahawalpur Cantt, Bahawalpur 63100, Pakistan; 4 School of Mathematics and Physics, Anhui Jianzhu University, Hefei 230601, China; 5 Department of Mathematics and Statistics, Quaid-e-Awam University of Engineering, Science and Technology, Sakrand Road, Nawabshah 67480, Pakistan; 6 Faculty of Education, Yuzuncu Yil University, 65090 Van, Turkey; 7 Department of Mathematics, Al-Qunfudah University College, Umm Al-Qura University, Mecca 24382, Saudi Arabia

**Keywords:** *C_6_H_6_* embedded in pyrene network, *C_6_H_6_* embedded in circumnaphthalene network, *C_6_H_6_* embedded in honeycomb network, *K*-Banhatti entropies

## Abstract

Entropy is a measure of a system’s molecular disorder or unpredictability since work is produced by organized molecular motion. Shannon’s entropy metric is applied to represent a random graph’s variability. Entropy is a thermodynamic function in physics that, based on the variety of possible configurations for molecules to take, describes the randomness and disorder of molecules in a given system or process. Numerous issues in the fields of mathematics, biology, chemical graph theory, organic and inorganic chemistry, and other disciplines are resolved using distance-based entropy. These applications cover quantifying molecules’ chemical and electrical structures, signal processing, structural investigations on crystals, and molecular ensembles. In this paper, we look at *K*-Banhatti entropies using *K*-Banhatti indices for $C_{6}H_{6}$ embedded in different chemical networks. Our goal is to investigate the valency-based molecular invariants and *K*-Banhatti entropies for three chemical networks: the circumnaphthalene ($CNB_{n}$), the honeycomb ($HB_{n}$), and the pyrene ($PY_{n}$). In order to reach conclusions, we apply the method of atom-bond partitioning based on valences, which is an application of spectral graph theory. We obtain the precise values of the first *K*-Banhatti entropy, the second *K*-Banhatti entropy, the first hyper *K*-Banhatti entropy, and the second hyper *K*-Banhatti entropy for the three chemical networks in the main results and conclusion.

## 1. Introduction

In the late 1990s, researchers began investigating the information content of complex networks, [1] and graphs based on Shannon’s entropy work [2]. Numerous quantitative measures for analyzing complex networks have been proposed [3,4] spanning a wide range of issues in discrete mathematics, computer science, information theory, statistics, chemistry, biology, and other fields [5,6]. Graph entropy measures, for example, have been widely used to characterize the structure of graph-based systems [7,8] in mathematical chemistry, biology, and computer science-related areas. The concept of graph entropy [9], developed by Rashevsky [10] and Trucco [11] has been used to quantify the structural complexity of graphs [12,13].

Chemical indices are important tools for studying different physico-chemical properties of molecules without having to conduct several tests. In the investigation of medicines, quantitative structure-activity relationships (QSAR) use mathematical computations to decipher the chemical properties [14,15]. Some researchers have analyzed the topological and *K*-Banhatti indices in [16,17]. Mowshowitz [18] introduced the entropy of a graph as an information-theoretic quantity. The complexity is evident here. The well-known Shannon’s entropy [2] is used to calculate the entropy of a graph. Importantly, Mowshowitz interpreted his graph entropy measure as a graph’s structural information content and demonstrated that this quantity satisfies important properties when used with product graphs [18]. Inspired by Dehmer and Kraus [19], it was discovered that determining the minimal values of graph entropies is difficult due to a lack of analytical methods to address this specific problem.

The first-order valence-based *K*-Banhatti indices [17,20,21] are, respectively, as follows:

(1) $$\;B_{1}\left(G,x\right)=\sum_{a_{i}∼a_{j}}x^{\left(V_{a_{i}}+V_{a_{j}}\right)}\;\;\;and\;\;B_{1_{\left(G\right)}}=\sum_{a_{i}∼a_{j}}\left(V_{a_{i}}+V_{a_{j}}\right).$$

where $a_{i}$ and $a_{j}$ denote the atoms of a molecule, $V_{a_{i}}$ and $V_{a_{j}}$ represent the valency of each atom, and, if $a_{i}$ and $a_{j}$ are connected or have atom bonds, then we denote this by $a_{i}∼a_{j}$. Accordingly, the second valence-based *K*-Banhatti index [22] and polynomial are as follows:

(2) $$\;B_{2}\left(G,x\right)=\sum_{a_{i}∼a_{j}}x^{\left(V_{a_{i}}\times V_{a_{j}}\right)}\;\;\;and\;\;B_{2_{\left(G\right)}}=\sum_{a_{i}∼a_{j}}\left(V_{a_{i}}\times V_{a_{j}}\right)$$

The hyper *K*-Banhatti index and first and second polynomial types [21] are as follows:

(3) $$\;HB_{1}\left(G,x\right)=\sum_{a_{i}∼a_{j}}x^{{\left(V_{a_{i}}+V_{a_{j}}\right)}^{2}}\;\;\;and\;\;HB_{1_{\left(G\right)}}=\sum_{a_{i}∼a_{j}}{\left(V_{a_{i}}+V_{a_{j}}\right)}^{2}$$

(4) $$\;HB_{2}\left(G,x\right)=\sum_{a_{i}∼a_{j}}x^{{\left(V_{a_{i}}\times V_{a_{j}}\right)}^{2}}\;\;\;and\;\;HB_{2_{\left(G\right)}}=\sum_{a_{i}∼a_{j}}{\left(V_{a_{i}}\times V_{a_{j}}\right)}^{2}$$

The Banhatti indices were proposed by the Indian mathematician Kulli as a result of Milan Randic’s 1972 Zagreb indices. With various techniques, such as modified and hyper-Banhatti indices, Kulli offered a number of studies on Banhatti indices. These indices have excellent associations with several chemical and nonchemical graph properties. The amount of thermal energy per unit temperature in a system that cannot be used for useful work is known as entropy [23,24]. In this article, we investigate the graphs of different molecules, namely the pyrene network, the circumnaphthalene series of benzenoid, and the honeycomb benzenoid network, to determine the *K*-Banhatti entropies’ use of *K*-Banhatti indices [21,25].

## 2. Definitions of Entropies via *K*-Banhatti Indices

Manzoor et al. in [26] and Ghani et al. in [27] recently offered another strategy that is a little bit novel in the literature: applying the idea of Shannon’s entropy [28] in terms of topological indices. The following formula represents the valency-based entropy:

(5) $$ENT_{\mu (G)}=-\sum_{a_{i}∼a_{j}}\frac{\mu (V_{a_{i}}V_{a_{j}})}{\sum_{a_{i}∼a_{j}}\mu \left(V_{a_{i}}V_{a_{j}}\right)}\log\{\frac{\mu (V_{a_{i}}V_{a_{j}})}{\sum_{a_{i}∼a_{j}}\mu \left(V_{a_{i}}V_{a_{j}}\right)}\}.$$

where $a_{1},a_{2}$ represents the atoms, $E_{G}$ represents the edge set, and $\mu (V_{a_{i}}V_{a_{j}})$ represents the edge weight of edge $\left(V_{a_{i}}V_{a_{j}}\right)$.

- **The first *K*-Banhatti entropy**

Let $\mu \left(V_{a_{i}}V_{a_{j}}\right)=V_{a_{i}}+V_{a_{j}}$. The first-order *K*-Banhatti index (1) is thus provided as

$$\begin{array}{ccc} B_{1_{\left(G\right)}} & = & \sum_{a_{i}∼a_{j}}\{V_{a_{i}}+V_{a_{j}}\}=\sum_{a_{i}∼a_{j}}\mu \left(V_{a_{i}}V_{a_{j}}\right). \end{array}$$

The use of (5), the first *K*-Banhatti entropy, is shown below:

(6) $$ENT_{B_{1_{\left(G\right)}}}=\log\left(B_{1_{\left(G\right)}}\right)-\frac{1}{B_{1_{\left(G\right)}}}\log\{{\prod_{a_{i}∼a_{j}}\left[V_{a_{i}}+V_{a_{j}}\right]}^{\left[V_{a_{i}}+V_{a_{j}}\right]}\}.$$

- **The Second *K*-Banhatti entropy**

Let $\mu \left(V_{a_{i}}V_{a_{j}}\right)=V_{a_{i}}\times V_{a_{j}}$. Then, the second *K*-Banhatti index (2) is given by

$$\begin{array}{c} B_{2_{\left(G\right)}}=\sum_{a_{i}∼a_{j}}\{\left(V_{a_{i}}\times V_{a_{j}}\right)\}=\sum_{a_{i}∼a_{j}}\mu \left(V_{a_{i}}V_{a_{j}}\right). \end{array}$$

The use of (5), the second *K*-Banhatti entropy, is shown below:

(7) $$ENT_{B_{2_{\left(G\right)}}}=\log\left(B_{2_{\left(G\right)}}\right)-\frac{1}{B_{2_{\left(G\right)}}}\log\{{\prod_{a_{i}∼a_{j}}\left[V_{a_{i}}\times V_{a_{j}}\right]}^{\left[V_{a_{i}}\times V_{a_{j}}\right]}\}.$$

- **Entropy related to the first *K*-hyper Banhatti index**

Let $\mu \left(V_{a_{i}}V_{a_{j}}\right)={\left(V_{a_{i}}+V_{a_{j}}\right)}^{2}$. Then, the first *K*-hyper Banhatti index (3) is given by

$$\begin{array}{c} HB_{1_{\left(G\right)}}=\sum_{a_{i}∼a_{j}}\{{\left(V_{a_{i}}+V_{a_{j}}\right)}^{2}\}=\sum_{a_{i}∼a_{j}}\mu \left(V_{a_{i}}V_{a_{j}}\right). \end{array}$$

The use of (5), the first *K*-hyper Banhatti entropy, is shown below:

(8) $$ENT_{HB_{1_{\left(G\right)}}}=\log\left(HB_{1_{\left(G\right)}}\right)-\frac{1}{HB_{1_{\left(G\right)}}}\log\{{\prod_{a_{i}∼a_{j}}\left[V_{a_{i}}+V_{a_{j}}\right]}^{2{\left[V_{a_{i}}+V_{a_{j}}\right]}^{2}}\}.$$

- **Entropy related to the second *K*-hyper Banhatti index**

Let $\mu \left(V_{a_{i}}V_{a_{j}}\right)={\left(V_{a_{i}}\times V_{a_{j}}\right)}^{2}$. Then, the second *K*-hyper Banhatti index (4) is given by

$$\begin{array}{ccc} HB_{2_{\left(G\right)}} & = & \sum_{a_{i}∼a_{j}}\{{\left(V_{a_{i}}\times V_{a_{j}}\right)}^{2}\}=\sum_{a_{i}∼a_{j}}\mu \left(V_{a_{i}}V_{a_{j}}\right). \end{array}$$

The use of (5), the second *K*-hyper Banhatti entropy, is shown below:

(9) $$ENT_{HB_{2_{\left(G\right)}}}=\log\left(HB_{1_{\left(G\right)}}\right)-\frac{1}{HB_{1_{\left(G\right)}}}\log\{{\prod_{a_{i}∼a_{j}}\left[V_{a_{i}}\times V_{a_{j}}\right]}^{2{\left[V_{a_{i}}\times V_{a_{j}}\right]}^{2}}\}.$$

## 3. The Pyrene Network

**Figure 1:**
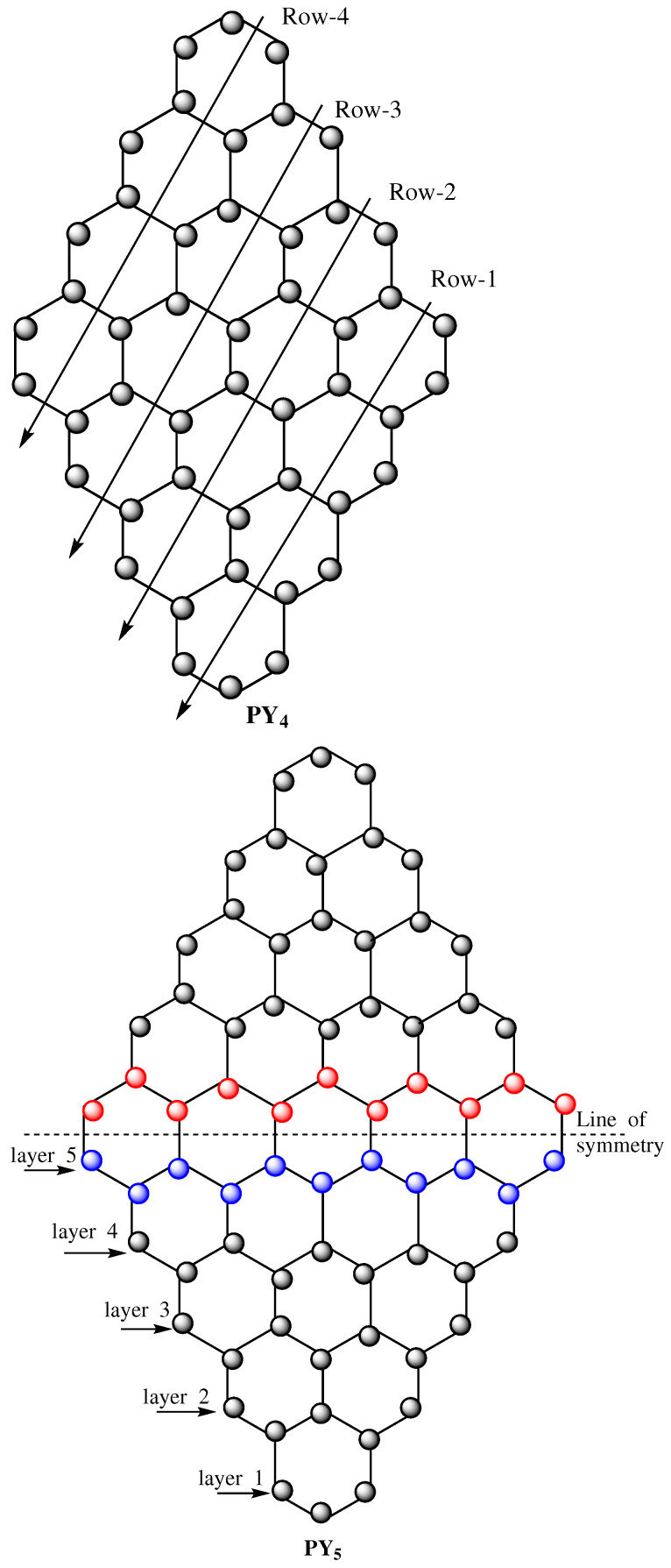
Pyrene network $PY_{n}$.

The precise arrangement of rings in the benzenoid system offers a transformation within a sequence of benzenoid structures of the benzenoid graph, which changes the structure. The Pyrene network $PY_{n}$ is a collection of hexagons, and it is a simple, connected 2D planner graph, where *n* represents the number of hexagons in any row of $PY_{n}$ (see Figure 1). Accordingly, the Pyrene network is a series of benzenoid rings, and the total number of benzenoid rings is $n^{2}$ in PY(n). We sum up the Zagreb polynomial and topological indices of PY(n) in this section.

**Results and discussion**

The number of atoms and the total number of atomic bonds for $PY_{n}$ are now determined. Let us consider the line of symmetry that divides $PY_{n}$ into two symmetric parts, as shown in Figure 1. Let us denote the number of atoms in one symmetric portion of $PY_{n}$ by *x* and the number of layers by *l*. In one symmetric part of $PY_{n}$, there are *l* layers of carbon atoms for 1≤l≤n, as indicated in Figure 1. Then, an *l*th layer contains 2l+1 carbon atoms. Accordingly, we have

$$\begin{array}{ccc} x & = & \sum_{l=1}^{n}\left(2l+1\right)=3+5+7+\ldots +\left(2n+1\right)\\ & = & \frac{n}{2}\{2\left(3\right)+\left(n-1\right)2\}\\ & = & n^{2}+2n\;\;\;\;\left(the\;\;sum\;\;of\;\;an\;\;arithmetic\;\;series\right) \end{array}$$

The number of atoms in $PY_{n}$ is $2x=2n^{2}+4n$ because of the two symmetric parts in $PY_{n}$. Furthermore, a $PY_{n}$ corner atom and an atom other than a corner atom have valencies two and three, respectively. Thus, out of $2n^{2}+4n$ atoms, 4n+2 atoms have valency two, and $2(n^{2}-1)$ atoms have valency three. So, by using Formula (1), the number of atomic bonds in $PY_{n}$ is $3n^{2}+4n-1$. According to the valencies (two and three) of the atoms, there are three types of atomic bonds, which are (2,2), (2,3), and (3,3) in $PY_{n}$. On the basis of valency, Table 1 provides the partition of the set of atomic bonds.

The edge partition of $PY_{n}$ is:

$$\begin{array}{ccc} V_{2∼2} & = & \{e=V_{i}∼V_{j},\;\;for\;all\;V_{i},V_{j}\;contained\;in\;E\left(PY_{n}\right),\;whenever\;d_{V_{i}}=2,d_{V_{j}}=2\},\\ V_{2∼3} & = & \{e=V_{i}∼V_{j},\;\;for\;all\;V_{i},V_{j}\;contained\;in\;E\left(PY_{n}\right),\;whenever\;d_{V_{i}}=2,d_{V_{j}}=3\},\\ V_{3∼3} & = & \{e=V_{i}∼V_{j},\;\;for\;all\;V_{i},V_{j}\;contained\;in\;E\left(PY_{n}\right),\;whenever\;d_{V_{i}}=3,d_{V_{j}}=3\}. \end{array}$$

This partition provides:

- **Entropy related to the first *K*-Banhatti index of $PY_{n}$**

Let $PY_{n}$ be the Pyrene network of $C_{6}H_{6}$. The first *K*-Banhatti polynomial is calculated using Equation (1) and Table 1.

(10) $$\begin{array}{ccc} B_{1}\left(PY_{n},x\right) & = & \sum_{V_{\left(2∼2\right)}}x^{2+2}+\sum_{V_{\left(2∼3\right)}}x^{2+3}+\sum_{V_{\left(3∼3\right)}}x^{3+3}\\ & = & 6x^{4}+8\left(n-1\right)x^{5}+\left(3n^{2}-4n+1\right)x^{6}. \end{array}$$

Following the simplification of Equation (10), we obtain the first *K*-Banhatti index, which is given at x=1 via differentiation.

(11) $$\begin{array}{c} B_{1}\left(PY_{n}\right)=2\left(9n^{2}+8n-5\right) \end{array}$$

Here, we calculate the first *K*-Banhatti entropy of $PY_{n}$ using Table 1 and Equation (11) inside Equation (6) in the following manner:

$$\begin{array}{ccc} ENT_{B_{1}}\left(PY_{n}\right) & = & \log\left(B_{1}\right)-\frac{1}{B_{1}}\log\{{\prod_{V_{\left(2,2\right)}}\left(V_{a_{i}}+V_{a_{j}}\right)}^{\left(V_{a_{i}}+V_{a_{j}}\right)}\times {\prod_{V_{\left(3,3\right)}}\left(V_{a_{i}}+V_{a_{j}}\right)}^{\left(V_{a_{i}}+V_{a_{j}}\right)}\\ & \times & {\prod_{V_{\left(3,3\right)}}\left(V_{a_{i}}+V_{a_{j}}\right)}^{\left(V_{a_{i}}+V_{a_{j}}\right)}\\ & = & \log2(9n^{2}+8n-5)-\frac{1}{29n^{2}+8n-5}\log\{16{\left(4\right)}^{4}\times 8\left(n-1\right){\left(5\right)}^{5}\times \left(3n^{2}-4n+1\right){\left(6\right)}^{6}. \end{array}$$

- **The second *K*-Banhatti entropy of $PY_{n}$**

Let $PY_{n}$ be the Pyrene network of $C_{6}H_{6}$. Then, using Equation (2) and Table 1, the second *K*-Banhatti polynomial is

(12) $$\begin{array}{ccc} B_{2}\left(PY_{n}\right) & = & \sum_{V_{\left(2∼2\right)}}x^{2\times 2}+\sum_{V_{\left(2∼3\right)}}x^{2\times 3}+\sum_{V_{\left(3∼3\right)}}x^{3\times 3}\\ & = & 6x^{4}+8\left(n-1\right)x^{6}+\left(3n^{2}-4n+1\right)x^{9}. \end{array}$$

To differentiate (34) at x=1, we obtain the second *K*-Banhatti index:

(13) $$\begin{array}{c} B_{2}\left(PY_{n}\right)=3\left(9n^{2}+4n-5\right). \end{array}$$

Here, we calculate the second *K*-Banhatti entropy of $PY_{n}$ using Table 1 and Equation (13) in Equation (7) as described below:

(14) $$\begin{array}{ccc} ENT_{B_{2}}\left(PY_{n}\right) & = & \log\left(B_{2}\right)-\frac{1}{B_{2}}\log\{{\prod_{V_{\left(2,2\right)}}\left(V_{a_{i}}\times V_{a_{j}}\right)}^{\left(V_{a_{i}}\times V_{a_{j}}\right)}\times {\prod_{V_{\left(2,3\right)}}\left(V_{a_{i}}\times V_{a_{j}}\right)}^{\left(V_{a_{i}}\times V_{a_{j}}\right)}\\ & \times & {\prod_{V_{\left(3,3\right)}}\left(V_{a_{i}}\times V_{a_{j}}\right)}^{\left(V_{a_{i}}\times V_{a_{j}}\right)}\\ & = & \log3(9n^{2}+4n-5)-\frac{1}{3(9n^{2}+4n-5)}\log\{16\left(6^{6}\right)\\ & \times & 8\left(n-1\right)9^{9}\times \left(3n^{2}-4n+1\right)12^{12}\times 2\left(2st-s-t\right)16^{16}\}. \end{array}$$

- **Entropy related to the first *K*-hyper Banhatti index of $PY_{n}$**

Let $PY_{n}$ be the Pyrene network of $C_{6}H_{6}$. Then, using Equation (3) and Table 1, the first *K*-hyper Banhatti polynomial is

(15) $$\begin{array}{ccc} HB_{1}\left(PY_{n}\right) & = & \sum_{V_{\left(2∼2\right)}}x^{{\left(2+2\right)}^{2}}+\sum_{V_{\left(2∼3\right)}}x^{{\left(2+3\right)}^{2}}+\sum_{V_{\left(3∼3\right)}}x^{{\left(3+3\right)}^{2}}\\ & = & 6x^{16}+8\left(n-1\right)x^{25}+\left(3n^{2}-4n+1\right)x^{36} \end{array}$$

To differentiate (15) at x=1, we obtain the first *K*-hyper Banhatti index

(16) $$\begin{array}{c} HB_{1}\left(PY_{n}\right)=2\left(54n^{2}+28n-34\right). \end{array}$$

Here, we calculate the first *K*-hyper Banhatti entropy of $PY_{n}$ using Table 1 and Equation (16) in Equation (9) as described below:

(17) $$\begin{array}{ccc} ENT_{HB_{1}}\left(PY_{n}\right) & = & \log\left(HB_{1}\right)-\frac{1}{HB_{1}}\log\{{\prod_{V_{\left(2,2\right)}}\left(V_{a_{i}}+V_{a_{j}}\right)}^{2{\left(V_{a_{i}}+V_{a_{j}}\right)}^{2}}\times {\prod_{V_{\left(2,3\right)}}\left(V_{a_{i}}+V_{a_{j}}\right)}^{2{\left(V_{a_{i}}+V_{a_{j}}\right)}^{2}}\\ & \times & {\prod_{V_{\left(3,3\right)}}\left(V_{a_{i}}+V_{a_{j}}\right)}^{2{\left(V_{a_{i}}+V_{a_{j}}\right)}^{2}}\\ & = & \log2(54n^{2}+28n-34)-\frac{1}{2(54n^{2}+28n-34)}\log\{16\left(5^{50}\right)\\ & \times & 8\left(n-1\right)\left(6^{72}\right)\times \left(3n^{2}-4n+1\right)\left(7^{98}\right)\times 2\left(2st-s-t\right)\left(8^{128}\right). \end{array}$$

- **Entropy related to the second *K*-hyper Banhatti index $PY_{n}$**

Let $PY_{n}$ be the Pyrene network of $C_{6}H_{6}$. Then, using Equation (4) and Table 1, the second *K*-hyper Banhatti polynomial is

(18) $$\begin{array}{ccc} HB_{2}\left(PY_{n}\right) & = & \sum_{V_{\left(2∼2\right)}}x^{{\left(2\times 2\right)}^{2}}+\sum_{V_{\left(2∼3\right)}}x^{{\left(2\times 3\right)}^{2}}+\sum_{V_{\left(3∼3\right)}}x^{{\left(3\times 3\right)}^{2}}\\ & = & 6x^{16}+8\left(n-1\right)x^{36}+\left(3n^{2}-4n+1\right)x^{81}. \end{array}$$

To differentiate (18) at x=1, we obtain the second *K*-hyper Banhatti index

(19) $$\begin{array}{c} HB_{2}\left(PY_{n}\right)=3\left(81n^{2}-12n-37\right). \end{array}$$

Here, we calculate the second *K*-hyper Banhatti entropy of $PY_{n}$ using Table 1 and Equation (19) in Equation (9) as described below:

(20) $$\begin{array}{ccc} ENT_{HB_{1}}\left(PY_{n}\right) & = & \log\left(HB_{1}\right)-\frac{1}{HB_{1}}\log\{{\prod_{V_{\left(2,2\right)}}\left(V_{a_{i}}\times V_{a_{j}}\right)}^{2{\left(V_{a_{i}}\times V_{a_{j}}\right)}^{2}}\times {\prod_{V_{\left(2,3\right)}}\left(V_{a_{i}}\times V_{a_{j}}\right)}^{2{\left(V_{a_{i}}\times V_{a_{j}}\right)}^{2}}\\ & \times & {\prod_{V_{\left(3,3\right)}}\left(V_{a_{i}}\times V_{a_{j}}\right)}^{2{\left(V_{a_{i}}\times V_{a_{j}}\right)}^{2}}\\ & = & \log3(81n^{2}-12n-37)-\frac{1}{3(81n^{2}-12n-37)}\log\{16{\left(6\right)}^{72}\\ & \times & 8\left(n-1\right)9^{81}\times \left(3n^{2}-4n+1\right)12^{288}\times 2\left(2st-s-t\right)16^{512} \end{array}$$

### Characteristics of *K*-Banhatti Indices of $PY_{n}$

**Figure 2:**
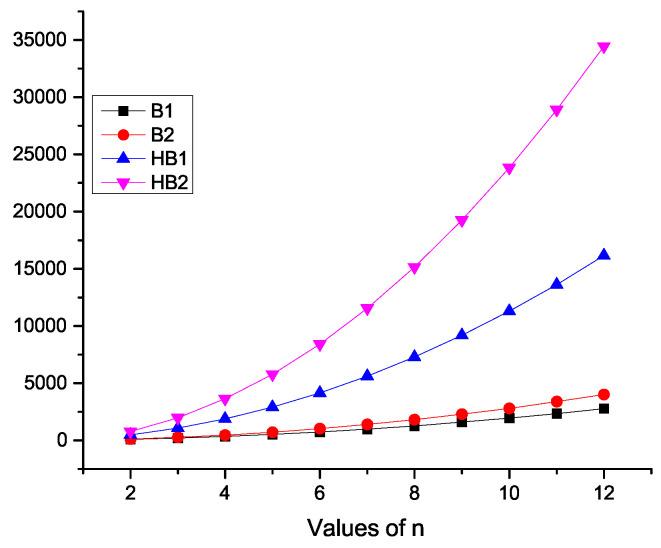

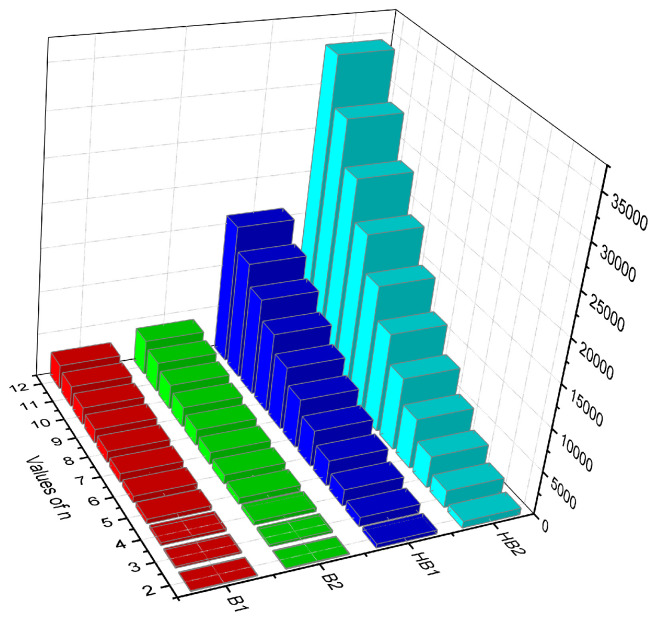
Graphical representation of *K*-Banhatti indices of $PY_{n}$.

Here, we contrast the *K*-Banhatti indices, namely $B_{1}$, $B_{2}$, $HB_{1}$, and $HB_{2}$ for $PY_{n}$ quantitatively and visually in Table 2 and Figure 2, respectively.

## 4. Circumnaphthalene Series of Benzenoid

**Figure 3:**
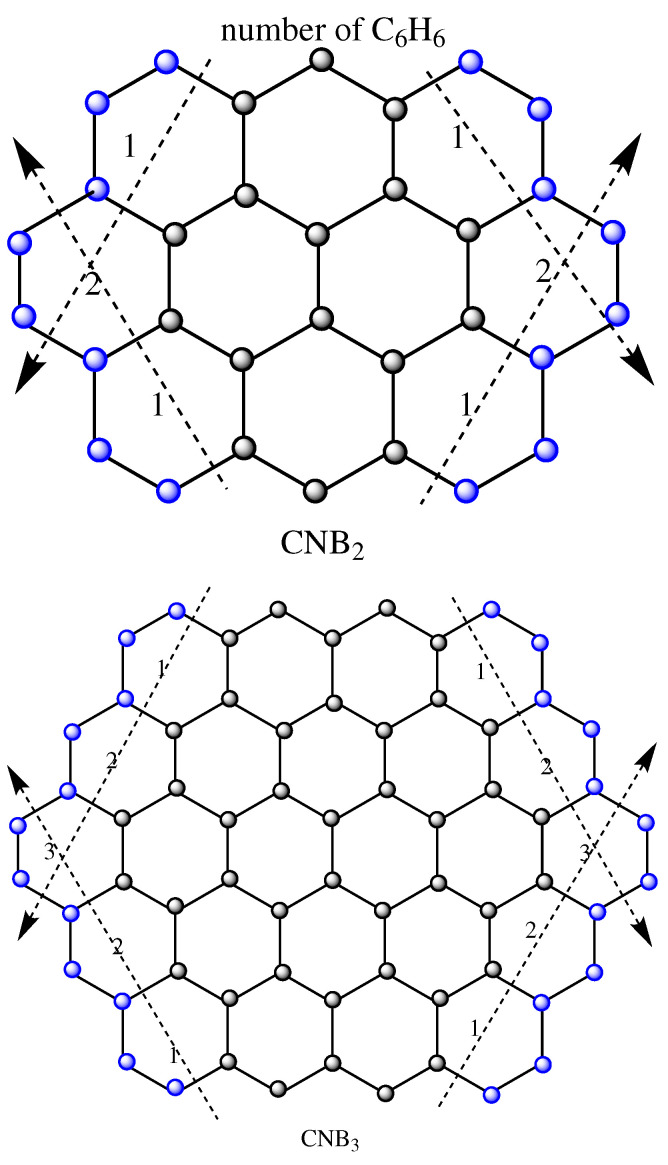
Circumnaphthalene series of benzenoid $CNB_{n}$.

Circumnaphthalene is similar to the benzenoid polycyclic aromatic hydrocarbons with the formula $C_{32}H_{14}$ and the ten peri-fused six-member rings in figure $CNB_{2}$. Ovalene is a chemical that is reddish-orange in color. It is only slightly soluble in solvents, such as benzenoid, toluene, and dichloromethane. The circumnaphthalene series of benzenoids is designated by $CNB_{n}$, where “*n*” is the number of benzenoid rings in the corner, as seen in Figure 3.

**Results and Discussion**

In Figure 3, we have the following three partitions of the carbon atoms in $CNB_{n}$:

$$\begin{array}{ccc} V_{2∼2} & = & \{V_{i}∼V_{j}=e,\forall \;V_{i},V_{j}\in E\left(CNB_{n}\right)|d_{CNB_{n}}\left(u\right)=2,d_{CNB_{n}}\left(v\right)=2\},\\ V_{2∼3} & = & \{V_{i}∼V_{j}=e,\forall \;V_{i},V_{j}\in E\left(CNB_{n}\right)|d_{CNB_{n}}\left(u\right)=2,d_{CNB_{n}}\left(v\right)=3\},\\ V_{3∼3} & = & \{V_{i}∼V_{j}=e,\forall \;V_{i},V_{j}\in E\left(CNB_{n}\right)|d_{CNB_{n}}\left(u\right)=3,d_{CNB_{n}}\left(v\right)=3\}. \end{array}$$

These partitions provide us with the atomic bond partition of the $CNB_{n}$ network (see Table 3).

- **Entropy related to the 1st *K*-Banhatti index of $CNB_{n}$**

Let $CNB_{n}$ be the circumnaphthalene series of benzenoid of $C_{6}H_{6}$. Then, using Equation (1) and Table 3, the first *K*-Banhatti polynomial is

(21) $$\begin{array}{ccc} B_{1}\left(CNB_{n},x\right) & = & \sum_{V_{\left(2∼2\right)}}x^{2+2}+\sum_{V_{\left(2∼3\right)}}x^{2+3}+\sum_{V_{\left(3∼3\right)}}x^{3+3}\\ & = & 6x^{4}+4\left(3n-5\right)x^{5}+\left(9n^{2}-27n+19\right)x^{6}. \end{array}$$

Following the simplification of Equation (21), we obtain the first *K*-Banhatti index, which is given at x=1 via differentiation.

(22) $$\begin{array}{c} B_{1}\left(CNB_{n}\right)=2\left(27n^{2}-51n+19\right). \end{array}$$

Here, we calculate the first *K*-Banhatti entropy of $CNB_{n}$ using Table 1 and Equation (24) in Equation (6) in the following manner:

$$\begin{array}{ccc} ENT_{B_{1}}\left(CNB_{n}\right) & = & \log\left(B_{1}\right)-\frac{1}{B_{1}}\log\{{\prod_{V_{\left(2,2\right)}}\left(V_{a_{i}}+V_{a_{j}}\right)}^{\left(V_{a_{i}}+V_{a_{j}}\right)}\times {\prod_{V_{\left(2,3\right)}}\left(V_{a_{i}}+V_{a_{j}}\right)}^{\left(V_{a_{i}}+V_{a_{j}}\right)}\\ & \times & {\prod_{V_{\left(3,3\right)}}\left(V_{a_{i}}+V_{a_{j}}\right)}^{\left(V_{a_{i}}+V_{a_{j}}\right)}\\ & = & \log2(27n^{2}-51n+19)-\frac{1}{2(27n^{2}-51n+19)}\log\{16{\left(4\right)}^{4}\\ & \times & 4\left(3n-5\right){\left(5\right)}^{5}\times \left(9n^{2}-27n+19\right){\left(6\right)}^{6}. \end{array}$$

- **The second *K*-Banhatti entropy of $CNB_{n}$**

Let $CNB_{n}$ be the circumnaphthalene series of benzenoid of $C_{6}H_{6}$. Then, using Equation (2) and Table 1, the second *K*-Banhatti polynomial is

(23) $$\begin{array}{ccc} B_{2}\left(CNB_{n}\right) & = & \sum_{V_{\left(2∼2\right)}}x^{2\times 2}+\sum_{V_{\left(2∼3\right)}}x^{2\times 3}+\sum_{V_{\left(3∼3\right)}}x^{3\times 3}\\ & = & 6x^{4}+4\left(3n-5\right)x^{6}+\left(9n^{2}-27n+19\right)x^{9}. \end{array}$$

To differentiate (23) at x=1, we obtain the second *K*-Banhatti index

(24) $$\begin{array}{c} B_{2}\left(CNB_{n}\right)=3\left(27n^{2}-57+25\right). \end{array}$$

Here, we calculate the second *K*-Banhatti entropy of $CNB_{n}$ using Table 3 and Equation (24) in Equation (7) as described below:

(25) $$\begin{array}{ccc} ENT_{B_{2}}\left(CNB_{n}\right) & = & \log\left(B_{2}\right)-\frac{1}{B_{2}}\log\{{\prod_{V_{\left(2,2\right)}}\left(V_{a_{i}}\times V_{a_{j}}\right)}^{\left(V_{a_{i}}\times V_{a_{j}}\right)}\times {\prod_{V_{\left(2,3\right)}}\left(V_{a_{i}}\times V_{a_{j}}\right)}^{\left(V_{a_{i}}\times V_{a_{j}}\right)}\\ & \times & {\prod_{V_{\left(3,3\right)}}\left(V_{a_{i}}\times V_{a_{j}}\right)}^{\left(V_{a_{i}}\times V_{a_{j}}\right)}\\ & = & \log3(27n^{2}-57+25)-\frac{1}{3(27n^{2}-57+25)}\log\{6\left(4^{4}\right)\\ & \times & 4\left(3n-5\right)6^{6}\times \left(9n^{2}-27n+19\right)9^{9}\}. \end{array}$$

- **Entropy related to the first K-hyper Banhatti index of ${\mathrm{CNB}}_{n}$**

Let $CNB_{n}$ be the circumnaphthalene series of benzenoid of $C_{6}H_{6}$. Then, using Equation (3) and Table 3, the first *K*-hyper Banhatti polynomial is

(26) $$\begin{array}{ccc} HB_{1}\left(CNB_{n}\right) & = & \sum_{V_{\left(2∼2\right)}}x^{{\left(2+2\right)}^{2}}+\sum_{V_{\left(2∼3\right)}}x^{{\left(2+3\right)}^{2}}+\sum_{V_{\left(3∼3\right)}}x^{{\left(3+3\right)}^{2}}\\ & = & 6x^{16}+4\left(3n-5\right)x^{25}+\left(9n^{2}-27n+19\right)x^{36} \end{array}$$

To differentiate (26) at x=1, we obtain the first *K*-hyper Banhatti index

(27) $$\begin{array}{c} HB_{1}\left(CNB_{n}\right)=4\left(81n^{2}-168n+70\right). \end{array}$$

Here, we calculate the first *K*-hyper Banhatti entropy of $CNB_{n}$ using Table 1 and Equation (27) in Equation (9) as described below:

(28) $$\begin{array}{ccc} ENT_{HB_{1}}\left(CNB_{n}\right) & = & \log\left(HB_{1}\right)-\frac{1}{HB_{1}}\log\{{\prod_{V_{\left(2,2\right)}}\left(V_{a_{i}}+V_{a_{j}}\right)}^{2{\left(V_{a_{i}}+V_{a_{j}}\right)}^{2}}\times {\prod_{V_{\left(2,3\right)}}\left(V_{a_{i}}+V_{a_{j}}\right)}^{2{\left(V_{a_{i}}+V_{a_{j}}\right)}^{2}}\\ & \times & {\prod_{V_{\left(3,3\right)}}\left(V_{a_{i}}+V_{a_{j}}\right)}^{2{\left(V_{a_{i}}+V_{a_{j}}\right)}^{2}}\\ & = & \log4(81n^{2}-168n+70)-\frac{1}{4(81n^{2}-168n+70)}\log\{6\left(4^{32}\right)\\ & \times & 4\left(3n-5\right)5^{50}\times \left(9n^{2}-27n+19\right)\left(6^{72}\right) \end{array}$$

- **Entropy related to the second *K*-hyper Banhatti index *CNB_n_***

Let $CNB_{n}$ be the circumnaphthalene series of benzenoid of $C_{6}H_{6}$. Then, using Equation (4) and Table 3, the second *K*-hyper Banhatti polynomial is

(29) $$\begin{array}{ccc} HB_{2}\left(CNB_{n}\right) & = & \sum_{V_{\left(2∼2\right)}}x^{{\left(2\times 2\right)}^{2}}+\sum_{V_{\left(2∼3\right)}}x^{{\left(2\times 3\right)}^{2}}+\sum_{V_{\left(3∼3\right)}}x^{{\left(3\times 3\right)}^{2}}\\ & = & 6x^{16}+4\left(3n-5\right)x^{36}+\left(9n^{2}-27n+19\right)x^{81}. \end{array}$$

To differentiate (29) at x=1, we obtain the second *K*-hyper Banhatti index

(30) $$\begin{array}{c} HB_{2}\left(CNB_{n}\right)=3\left(243n^{2}-585n+305\right). \end{array}$$

Here, we calculate the second *K*-hyper Banhatti entropy of $CNB_{n}$ using Table 3 and Equation (30) in Equation (9) as described below:

(31) $$\begin{array}{ccc} ENT_{HB_{2}}\left(CNB_{n}\right) & = & \log\left(HB_{2}\right)-\frac{1}{HB_{2}}\log\{{\prod_{V_{\left(2,2\right)}}\left(V_{a_{i}}\times V_{a_{j}}\right)}^{2{\left(V_{a_{i}}\times V_{a_{j}}\right)}^{2}}\times {\prod_{V_{\left(2,3\right)}}\left(V_{a_{i}}\times V_{a_{j}}\right)}^{2{\left(V_{a_{i}}\times V_{a_{j}}\right)}^{2}}\\ & \times & {\prod_{V_{\left(3,3\right)}}\left(V_{a_{i}}\times V_{a_{j}}\right)}^{2{\left(V_{a_{i}}\times V_{a_{j}}\right)}^{2}}\\ & = & \log3(243n^{2}-585n+305)-\frac{1}{3(243n^{2}-585n+305)}\log\{6{\left(4\right)}^{32}\\ & \times & 4\left(3n-5\right)6^{72}\times \left(9n^{2}-27n+19\right)9^{162} \end{array}$$

### Characteristics of *K*-Banhatti Indices of $CNB_{n}$

**Figure 4:**
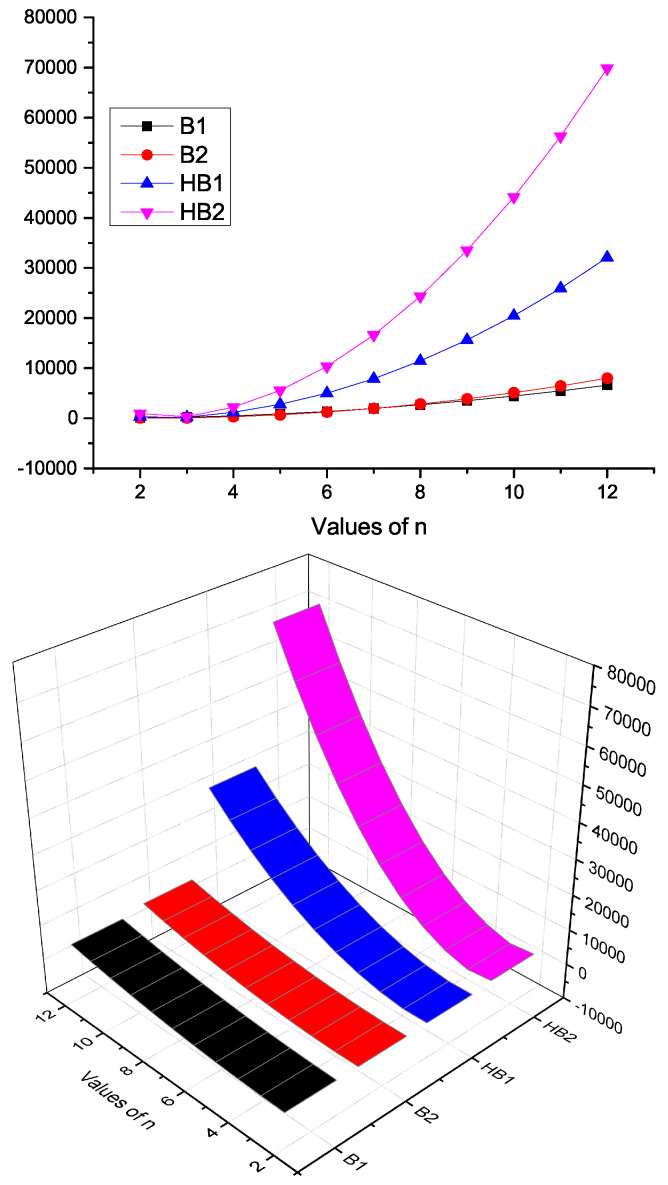
Graphical representation of *K*-Banhatti indices of $CNB_{n}$.

Here, we contrast the *K*-Banhatti indices, namely $B_{1}$, $B_{2}$, $HB_{1}$, and $HB_{2}$ for $CNB_{n}$ quantitatively and visually in Table 4 and Figure 4, respectively.

## 5. The Honeycomb Benzenoid Network

**Figure 5:**
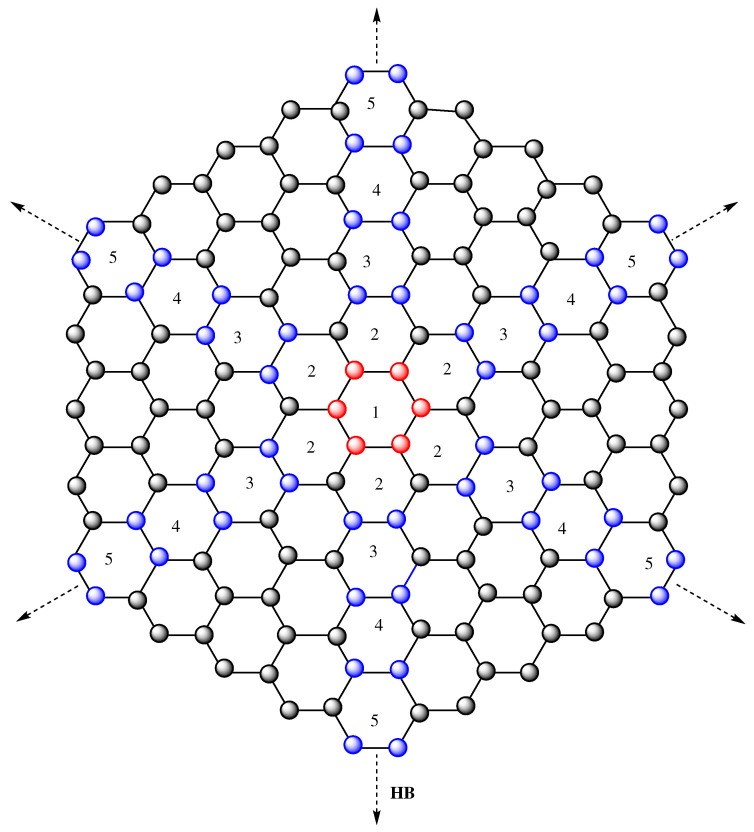
The honeycomb benzenoid network.

In this section, we introduce a chemical compound that has received more and more attention in recent years, partly due to its applications in chemistry. Honeycomb networks are formed when hexagonal tiling is used recursively in a specific pattern. $HB_{n}$ denotes an *n*-dimensional honeycomb network, where *n* is the number of Benzene rings from center to top, center to bottom, or center to each corner of $HB_{n}$, as shown in Figure 5.

**Results and Discussion**

The honeycomb network $HB_{n}$ is created by adding a layer of hexagons around the boundary of $HB_{\left(n-1\right)}$. In the honeycomb benzenoid network, a 6n amount of atoms has valency two, and $6n^{2}-6n$ atoms have valency three. According to the valency of each atom in $HB_{n}$, the atomic bonds are classified into three types: 2∼2, 2∼3, and 3∼3 (see Figure 5).

$$\begin{array}{ccc} E_{G_{2∼2}} & = & \{e=u∼v,\forall \;u,v\in E\left(HB_{n}\right)|d_{u}=2,d_{v}=2\},\\ E_{G_{2∼3}} & = & \{e=u∼v,\forall \;u,v\in E\left(HB_{n}\right)|d_{u}=2,d_{v}=3\},\\ E_{G_{3∼3}} & = & \{e=u∼v,\forall \;u,v\in E\left(HB_{n}\right)|d_{u}=3,d_{v}=3\}. \end{array}$$

Thus, according to the above partition of the atomic bonds, there is 3n(3n−1) total number of atomic bonds used in the honeycomb benzenoid network. The atomic bond partition of $HB_{n}$ is shown in Table 5.

- **Entropy related to the first *K*-Banhatti index of $HB_{n}$**

Let $HB_{n}$ be the honeycomb benzenoid network of $C_{6}H_{6}$. Then, using Equation (1) and Table 5, the first *K*-Banhatti polynomial is

(32) $$\begin{array}{ccc} B_{1}\left(HB_{n},x\right) & = & \sum_{V_{\left(2∼2\right)}}x^{2+2}+\sum_{V_{\left(2∼3\right)}}x^{2+3}+\sum_{V_{\left(3∼3\right)}}x^{3+3}\\ & = & 6x^{4}+12\left(n-1\right)x^{5}+\left(9n^{2}-15n+6\right)x^{6}. \end{array}$$

Following the simplification of Equation (32), we obtain the first *K*-Banhatti index given at x=1 via differentiation.

(33) $$\begin{array}{c} B_{1}\left(HB_{n}\right)=2\left(27n^{2}-15n-26\right). \end{array}$$

Here, we calculate the first *K*-Banhatti entropy of $HB_{n}$ using Table 5 and Equation (33) in Equation (6) in the following manner:

$$\begin{array}{ccc} ENT_{B_{1}}\left(HB_{n}\right) & = & \log\left(B_{1}\right)-\frac{1}{B_{1}}\log\{{\prod_{V_{\left(2,2\right)}}\left(V_{a_{i}}+V_{a_{j}}\right)}^{\left(V_{a_{i}}+V_{a_{j}}\right)}\times {\prod_{V_{\left(2,3\right)}}\left(V_{a_{i}}+V_{a_{j}}\right)}^{\left(V_{a_{i}}+V_{a_{j}}\right)}\\ & \times & {\prod_{V_{\left(3,3\right)}}\left(V_{a_{i}}+V_{a_{j}}\right)}^{\left(V_{a_{i}}+V_{a_{j}}\right)}\\ & = & \log2(27n^{2}-15n-26)-\frac{1}{2(27n^{2}-15n-26)}\log\{6{\left(4\right)}^{4}\\ & \times & 12\left(n-1\right){\left(5\right)}^{5}\times \left(9n^{2}-15n+6\right){\left(6\right)}^{6}. \end{array}$$

- **The second *K*-Banhatti entropy of $HB_{n}$**

Let $HB_{n}$ be the honeycomb benzenoid network of $C_{6}H_{6}$. Then, using Equation (2) and Table 5, the second *K*-Banhatti polynomial is

(34) $$\begin{array}{ccc} B_{2}\left(HB_{n}\right) & = & \sum_{V_{\left(2∼2\right)}}x^{2\times 2}+\sum_{V_{\left(2∼3\right)}}x^{2\times 3}+\sum_{V_{\left(3∼3\right)}}x^{3\times 3}\\ & = & 6x^{4}+12\left(n-1\right)x^{6}+\left(9n^{2}-15n+6\right)x^{9}. \end{array}$$

To differentiate (34) at x=1, we obtain the second *K*-Banhatti index

(35) $$\begin{array}{c} B_{2}\left(HB_{n}\right)=81n^{2}-87n+30. \end{array}$$

Here, we calculate the second *K*-Banhatti entropy of $HB_{n}$ using Table 5 and Equation (35) in Equation (7) as described below

(36) $$\begin{array}{ccc} ENT_{B_{2}}HB_{n} & = & \log\left(B_{2}\right)-\frac{1}{B_{2}}\log\{{\prod_{V_{\left(2,2\right)}}\left(V_{a_{i}}\times V_{a_{j}}\right)}^{\left(V_{a_{i}}\times V_{a_{j}}\right)}\times {\prod_{V_{\left(2,3\right)}}\left(V_{a_{i}}\times V_{a_{j}}\right)}^{\left(V_{a_{i}}\times V_{a_{j}}\right)}\\ & \times & {\prod_{V_{\left(3,3\right)}}\left(V_{a_{i}}\times V_{a_{j}}\right)}^{\left(V_{a_{i}}\times V_{a_{j}}\right)}\\ & = & \log\left(81n^{2}-87n+30\right)-\frac{1}{81n^{2}-87n+30}\log\{6\left(4^{4}\right)\\ & \times & 12\left(n-1\right)6^{6}\times \left(9n^{2}-15n+6\right)9^{9}\}. \end{array}$$

- **Entropy related to the first *K*-hyper Banhatti index of $HB_{n}$**

Let $HB_{n}$ be the honeycomb benzenoid network of $C_{6}H_{6}$. Then, using Equation (3) and Table 5, the first *K*-hyper Banhatti polynomial is

(37) $$\begin{array}{ccc} HB_{1}\left(HB_{n}\right) & = & \sum_{V_{\left(2∼2\right)}}x^{{\left(2+2\right)}^{2}}+\sum_{V_{\left(2∼3\right)}}x^{{\left(2+3\right)}^{2}}+\sum_{V_{\left(3∼3\right)}}x^{{\left(3+3\right)}^{2}}\\ & = & 6x^{16}+12\left(n-1\right)x^{25}+\left(9n^{2}-15n+6\right)x^{36} \end{array}$$

To differentiate (37) at x=1, we obtain the first *K*-hyper Banhatti index

(38) $$\begin{array}{c} HB_{1}\left(HB_{n}\right)=12\left(27n^{2}-20n+1\right). \end{array}$$

Here, we calculate the first *K*-hyper Banhatti entropy of $HB_{n}$ using Table 5 and Equation (38) in Equation (9) as described below:

(39) $$\begin{array}{ccc} ENT_{HB_{1}}HB_{n} & = & \log\left(HB_{1}\right)-\frac{1}{HB_{1}}\log\{{\prod_{V_{\left(2,2\right)}}\left(V_{a_{i}}+V_{a_{j}}\right)}^{2{\left(V_{a_{i}}+V_{a_{j}}\right)}^{2}}\times {\prod_{V_{\left(2,3\right)}}\left(V_{a_{i}}+V_{a_{j}}\right)}^{2{\left(V_{a_{i}}+V_{a_{j}}\right)}^{2}}\\ & \times & {\prod_{V_{\left(3,3\right)}}\left(V_{a_{i}}+V_{a_{j}}\right)}^{2{\left(V_{a_{i}}+V_{a_{j}}\right)}^{2}}\\ & = & \log12(27n^{2}-20n+1)-\frac{1}{12(27n^{2}-20n+1)}\log\{6\left(4^{32}\right)\\ & \times & 12\left(n-1\right)\left(5^{50}\right)\times \left(9n^{2}-15n+6\right)\left(6^{72}\right)\}. \end{array}$$

- **Entropy related to the second *K*-hyper Banhatti index $HB_{n}$**

Let $HB_{n}$ be the honeycomb benzenoid network of $C_{6}H_{6}$. Then, using Equation (4) and Table 5, the second *K*-hyper Banhatti polynomial is

(40) $$\begin{array}{ccc} HB_{2}\left(HB_{n}\right) & = & \sum_{V_{\left(2∼2\right)}}x^{{\left(2\times 2\right)}^{2}}+\sum_{V_{\left(2∼3\right)}}x^{{\left(2\times 3\right)}^{2}}+\sum_{V_{\left(3∼3\right)}}x^{{\left(3\times 3\right)}^{2}}\\ & = & 6x^{4}+12\left(n-1\right)x^{36}+\left(9n^{2}-15n+6\right)x^{81}. \end{array}$$

To differentiate (40) at x=1, we obtain the second *K*-hyper Banhatti index

(41) $$\begin{array}{c} HB_{2}\left(HB_{n}\right)=3\left(243n^{2}-261n+26\right). \end{array}$$

Here, we calculate the second *K*-hyper Banhatti entropy of $HB_{n}$ using Table 5 and Equation (41) in Equation (9), as described below:

(42) $$\begin{array}{ccc} ENT_{HB_{1}}HB_{n} & = & \log\left(HB_{1}\right)-\frac{1}{HB_{1}}\log\{{\prod_{V_{\left(2,2\right)}}\left(V_{a_{i}}\times V_{a_{j}}\right)}^{2{\left(V_{a_{i}}\times V_{a_{j}}\right)}^{2}}\times {\prod_{V_{\left(2,3\right)}}\left(V_{a_{i}}\times V_{a_{j}}\right)}^{2{\left(V_{a_{i}}\times V_{a_{j}}\right)}^{2}}\\ & \times & {\prod_{V_{\left(3,3\right)}}\left(V_{a_{i}}\times V_{a_{j}}\right)}^{2{\left(V_{a_{i}}\times V_{a_{j}}\right)}^{2}}\\ & = & \log3(243n^{2}-261n+26)-\frac{1}{3(243n^{2}-261n+26)}\log\{6{\left(4\right)}^{32}\\ & \times & 12\left(n-1\right)6^{72}\times \left(9n^{2}-15n+6\right)9^{162}. \end{array}$$

### Characteristics of *K*-Banhatti Indices of $HB_{n}$

**Figure 6:**
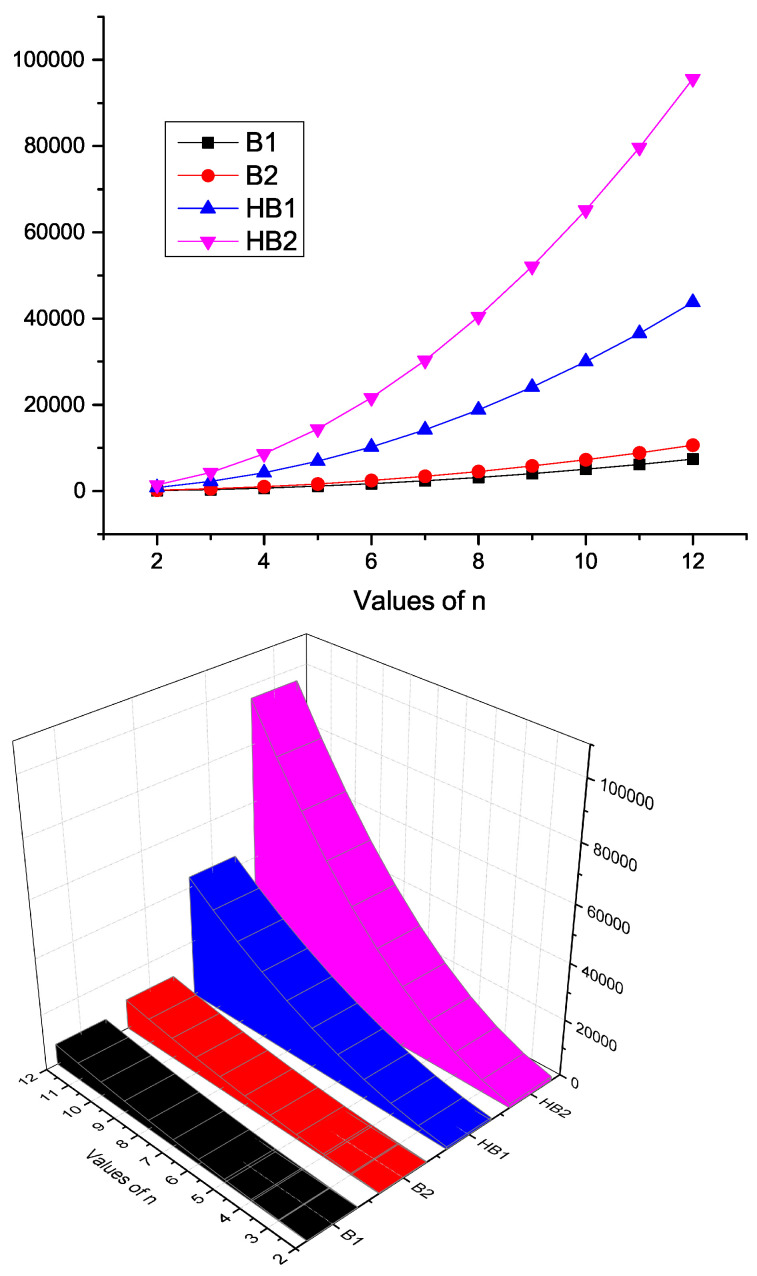
Graphical representation of *K*-Banhatti indices of $HB_{n}$.

Here, we contrast the *K*-Banhatti indices, namely $B_{1}$, $B_{2}$, $HB_{1}$, and $HB_{2}$ for $HB_{n}$ quantitatively and visually in Table 6 and Figure 6, respectively.

## 6. Conclusions

By using Shannon’s entropy and the entropy definitions of Chen et al., we looked into the graph entropies connected to a novel information function and assessed the link between degree-based topological indices and degree-based entropies in this work. Industrial chemistry has a strong foundation in the concept of distance-based entropy. The Pyrene network, $PY_{n}$; the circumnaphthalene series of benzenoid, $CNB_{n}$; and the honeycomb benzenoid network, $HB_{n}$ were studied, and their valency-based *K*-Banhatti indices were estimated using four *K*-Banhatti polynomials with a set partition and an atom bonds approach. The acquired results are valuable for anticipating numerous molecular features of chemical substances, such as boiling point, π electron energy, pharmaceutical configuration, and many more concepts. Our results can be applied to determine the electronic structure, signal processing, physicochemical reactions, and complexity of molecules and molecular ensembles for $PY_{n}$, $CNB_{n}$, and $HB_{n}$. Together with chemical structure, thermodynamic entropy, energy, and computer sciences, the *K*-Banhatti entropy can be crucial to linking different fields and serving as the basis for future interdisciplinary research. We intend to extend this idea to different chemical structures in the future, opening up new directions for study in this area.

## Figures and Tables

**Table 1:** Atomic bond partition of $PY_{n}$.

Atomic Bond Type	$V_{2∼2}$	$V_{2∼3}$	$V_{3∼3}$
Number of atom bonds	6	8(n−1)	$3n^{2}-4n+1$

**Table 2:** Numerical values of *K*-Banhatti indices of $PY_{n}$.

Values of n	2	3	4	5	6	7	8	9	10	11	12
$B_{1}$	94	200	342	520	734	984	1270	1592	1950	2344	2774
$B_{2}$	117	264	465	720	1029	1392	1809	2280	2805	3384	4017
$HB_{1}$	476	1072	1884	2912	4156	5616	7292	9184	11,292	13,616	16,156
$HB_{2}$	789	1968	3633	5784	8421	11,544	15,153	19,248	23,829	28,896	34,449

**Table 3:** Atomic bond partition of $CNB_{n}$ network.

Types of Atomic Bond	$V_{2∼2}$	$V_{2∼3}$	$V_{3∼3}$
$m_{V_{i}∼V_{j}}$	6	4(3n−5)	$9n^{2}-27n+19$

**Table 4:** Numerical comparison of topological indices of $CNB_{n}$.

Values of n	2	3	4	5	6	7	8	9	10	11	12
$B_{1}$	50	218	494	8787	1370	1970	2678	3494	4418	5450	6590
$B_{2}$	75	57	291	678	1245	1965	2847	3891	5097	6465	7995
$HB_{1}$	280	232	1180	2776	5020	7812	11,452	15,640	20,476	25,960	32,092
$HB_{2}$	915	321	2211	5559	10,365	16,629	24,351	33,531	44,169	56,265	69,819

**Table 5:** Atomic bond partition of $HB_{n}$.

Types of Atomic Bonds	$E_{G_{2∼2}}$	$E_{G_{2∼3}}$	$E_{G_{3∼3}}$
Cardinality of atomic bonds	6	12(n−1)	$\left(9n^{2}-15n+6\right)$

**Table 6:** Numerical comparison of topological indices of $HB_{n}$.

Values of n	2	3	4	5	6	7	8	9	10	11	12
$B_{1}$	104	344	692	1148	1712	2384	3164	4052	5048	6152	7364
$B_{2}$	180	498	978	1620	2424	3390	4518	5808	7260	8874	10650
$HB_{1}$	828	2208	4236	6912	10,236	14,208	18,828	24,096	30,012	36,576	43,788
$HB_{2}$	1428	4290	8610	14,388	21,624	30,318	40,470	52,080	65,148	79,674	95,658

## Data Availability

No data available to support this study.

## Sample Availability

No data available.
